# Production of cinnamic and *p*-hydroxycinnamic acid from sugar mixtures with engineered *Escherichia coli*

**DOI:** 10.1186/s12934-014-0185-1

**Published:** 2015-01-16

**Authors:** Alejandra Vargas-Tah, Luz María Martínez, Georgina Hernández-Chávez, Mario Rocha, Alfredo Martínez, Francisco Bolívar, Guillermo Gosset

**Affiliations:** Departamento de Ingeniería Celular y Biocatálisis, Universidad Nacional Autónoma de México, Apdo. Postal 510-3, Cuernavaca, Morelos 62210 México; Departamento de Biología Molecular de Plantas, Instituto de Biotecnología, Universidad Nacional Autónoma de México, Apdo. Postal 510-3, Cuernavaca, Morelos 62210 México

**Keywords:** *Escherichia coli*, Aromatics, *P-*hydroxycinnamic acid, Cinnamic acid, Phosphoenolpyruvate: carbohydrate phosphotransferase system, Sugar mixtures, Metabolic engineering

## Abstract

**Background:**

The aromatic compounds cinnamic acid (CA) and *p*-hydroxycinnamic acid (pHCA) are used as flavoring agents as well as precursors of chemicals. These compounds are present in plants at low concentrations, therefore, complex purification processes are usually required to extract the product. An alternative production method for these aromatic acids is based on the use of microbial strains modified by metabolic engineering. These biotechnological processes are usually based on the use of simple sugars like glucose as a raw material. However, sustainable production processes should preferably be based on the use of waste material such as lignocellulosic hydrolysates.

**Results:**

In this study, *E. coli* strains with active (W3110) and inactive phosphoenolpyruvate:sugar phosphotransferase system (PTS) (VH33) were engineered for CA and pHCA production by transforming them with plasmids expressing genes encoding phenylalanine/tyrosine ammonia lyase (PAL/TAL) enzymes from *Rhodotorula glutinis* or *Arabidopsis thaliana* as well as genes *aroG*^*fbr*^ and *tktA*, encoding a feedback inhibition resistant version of 3-deoxy-D-*arabino*-heptulosonate 7-phosphate synthase and transketolase, respectively. The generated strains were evaluated in cultures with glucose, xylose or arabinose, as well as a simulated lignocellulosic hydrolysate containing a mixture of these three sugars plus acetate. Production of CA was detected in strains expressing PAL/TAL from *A. thaliana*, whereas both CA and pHCA accumulated in strains expressing the enzyme from *R. glutinis*. These experiments identified arabinose and W3110 expressing PAL/TAL from *A. thaliana*, *aroG*^*fbr*^ and *tktA* as the carbon source/strain combination resulting in the best CA specific productivity and titer. To improve pHCA production, a mutant with inactive *pheA* gene was generated, causing an 8-fold increase in the yield of this aromatic acid from the sugars in a simulated hydrolysate.

**Conclusions:**

In this study the quantitative contribution of active or inactive PTS as well as expression of PAL/TAL from *R. glutinis* or *A. thaliana* were determined for production performance of CA and pHCA when growing on carbon sources derived from lignocellulosic hydrolysates. These data will be a useful resource in efforts towards the development of sustainable technologies for the production of aromatic acids.

**Electronic supplementary material:**

The online version of this article (doi:10.1186/s12934-014-0185-1) contains supplementary material, which is available to authorized users.

## Introduction

The phenylpropanoid pathway is a natural source for a diverse group of secondary metabolites having applications as nutraceutical and pharmaceutical agents as well as chemical precursors [1]. Two examples of valuable phenylpropanoids are cinnamic acid (CA) and *p*-hydroxycinnamic acid (pHCA). Applications of CA include its use as flavoring agent and as an anti-bacterial compound, whereas pHCA is a precursor for chemicals employed in the generation of thermoplastics, health, cosmetic and flavoring products [2,3]. Both aromatic acids can be obtained from plant extracts, however, these compounds are found at low concentrations in plant tissues, resulting in low yields and/or low purity of the final products. To circumvent this limitation, alternative production methods are being evaluated. The biotechnological production of phenylpropanoids is now possible as a result of the application of metabolic engineering to suitable microbial hosts [4-6]. This development has enabled the possibility of large-scale industrial production of CA, pHCA and derived compounds from simple carbon sources [3].

**Figure 1:**
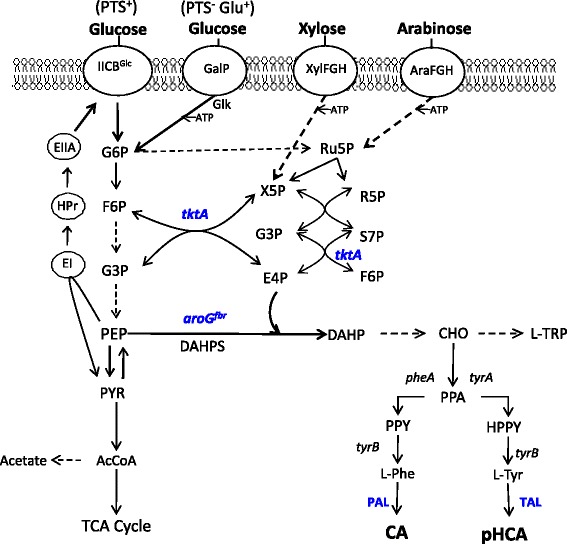
**Central metabolism, sugar import routes and aromatics biosynthetic pathways.** Dashed arrows indicate multiple enzyme reactions. EI, enzyme I; HPr, phosphohistidine carrier protein; EIIA, glucose-specific enzyme II; IICB^Glc^, integral membrane glucose permease; GalP, galactose permease; XylFGH, xylose transport proteins, AraFGH, arabinose transport proteins; G6P, glucose-6-phosphate; F6P, fructose-6-phosphate; G3P, glyceraldehyde-3-phosphate; PEP, phosphoenolpyruvate; R5P, ribose-5-phosphate; Ru5P, ribulose-5-phosphate; S7P, sedoheptulose-7-phosphate; X5P, xylulose-5-phosphate; PYR, pyruvate; AcCoA, acetyl-CoA; TCA, tricarboxylic acid; *aroG* ^*fbr*^, gene encoding a feedback-inhibition-resistant version of 3-deoxy-D-*arabino*-heptulosonate-7-phosphate synthase (DAHPS); *tktA*, transketolase; *tyrB*, tyrosine aminotransferase gene; PAL, phenylalanine ammonia lyase; TAL, tyrosine ammonia lyase.

The general strategy followed for the generation of microbial strains with the capacity to synthesize CA or pHCA from a simple carbon source involves performing genetic modifications that result in increased carbon flow to the L-Phe or L-Tyr biosynthetic pathways, respectively. In most bacteria and plants, the common aromatic pathway starts with the condensation of phosphoenolpyruvate (PEP) and erythrose-4-phosphate (E4P) to yield 3-deoxy-D-*arabino*-heptulosonate-7-phosphate (DAHP) (Figure 1). After seven enzymatic reactions, chorismate (CHO), a common precursor for L-Phe, L-Tyr and L-tryptophan biosynthesis, is synthesized. A frequent strategy employed to increase carbon flow from central metabolism to the common aromatic pathway involves the expression of a feedback-inhibition-resistant (fbr) mutant version of enzyme DAHP synthase (DAHPS) [7]. This modification causes an increased rate of CHO synthesis. Increasing carbon flow from CHO to L-Phe biosynthesis involves the expression of a feedback-inhibition-resistant version of enzyme CHO mutase-prephenate dehydratase (CM-PDT), whereas a similar approach is followed for increasing L-Tyr synthesis by expressing a feedback-inhibition-resistant version of CHO mutase-prephenate dehydrogenase (CM-PDH) (Figure 1) [8-10]. Further improvement of L-Phe and L-Tyr production strains can be achieved by increasing availability of precursors PEP and erythrose-4-phosphate (E4P). The overexpression of genes *ppsA* encoding PEP synthetase (Pps) and *tktA* encoding transketolase, and enzyme involved in E4P synthesis, has resulted in increased L-Tyr synthesis capacity [11]. An alternate strategy to increase PEP availability for aromatics synthesis involves the generation of *E. coli* strains lacking the PEP:sugar phosphotransferase system (PTS) activity, and further modified to display a high growth rate on glucose (PTS^−^ glucose^+^ phenotype). Growth of this type of mutant strains on glucose is dependent on transport by galactose permease (GalP) and ATP-dependent phosphorylation by glucokinase [12]. Since PEP is not consumed during glucose import, these PTS^−^ glucose^+^ strains display a higher aromatics yield from glucose when compared to a wild type PTS^+^ strain [13].

The biological synthesis of CA and pHCA proceeds by deamination of L-phenylalanine (L-Phe) and L-tyrosine (L-Tyr), respectively. These reactions are catalyzed by enzymes having phenylalanine ammonia-lyase (PAL) and tyrosine ammonia-lyase (TAL) activities [14-16]. It should be noted that enzymes having TAL activity also display activity with L-Phe as substrate, at present, no enzyme having only TAL activity has been reported [5]. The affinity for L-Phe or L-Tyr varies among different TAL enzymes [15,16]. For the above reasons, these proteins are usually designated as PAL/TAL enzymes to indicate specificity towards both substrates. In this study, the enzyme PAL/TAL from *R. glutinis* was used for pHCA production since this enzyme shows more affinity towards L-Tyr than L-Phe [16,17]. In addition, the PAL2 isoform from *A. thaliana* was also selected as model enzyme for production of CA. Four PAL isoforms have been described for *A. thaliana*, the PAL1 isoform has been used to synthesize CA as intermediate in naringenin production [17,18]. In our study, PAL2 was selected because the Km (64 μM) for L-Phe is lower when compared to the other three isoforms [19].

Several microbial species have been engineered for the production of CA and pHCA from simple carbon sources. *E. coli* strains modified for L-Phe or L-Tyr overproduction have been further engineered for the production of CA or pHCA by the heterologous expression of enzymes having PAL/TAL activities. A screening for enzymes having PAL/TAL activity identified an enzyme from the yeast *R. glutinis* as having a high level of TAL activity. The gene coding for this enzyme was cloned and expressed in *E. coli* and *Saccharomyces cerevisiae* strains with other metabolic modifications, resulting in the synthesis of pHCA from glucose [5]. Expression of the *PAL* gene from the yeast *R. glutinis* in an *E. coli* strain that overproduces L-Phe resulted in the production of both CA (~380 μM) and pHCA (~150 μM) from glucose [5]. An *E. coli* strain for pHCA production was generated by expression of fbr versions of DAHP synthase, CM-PDH as well as a codon-optimized version of the *tal* gene coding for a TAL from the actinomycete *Saccharothrix espanaensis*. The engineered *E. coli* strain produced 974 mg/L pHCA from glucose in 36 h [20]. The solvent-tolerant bacterium *Pseudomonas putida* S12 has been engineered by expression of the *pal* gene from the yeast *Rhodosporidium toruloides* and other metabolic engineering modifications to produce CA and pHCA from glucose with titers of 5 and 10.6 mM, respectively [6,21].

Previous examples illustrate the feasibility of producing CA and pHCA from a simple carbon source such as glucose. This is an important step towards replacing current chemical synthesis for the production of these aromatic acids. However, improvement of biotechnological production processes is still feasible. The utilization as raw material of carbon sources directly derived from biomass, such as sugar polymers or sugar mixtures constitute a step forward towards production process sustainability. Production of CA from biomass-derived carbon sources such as starch or cellulose has been reported with *Streptomyces lividans*. The expression of a gene coding for an enzyme with PAL activity from *Streptomyces maritimus* in *S. lividans* resulted in a strain with the capacity to synthesize CA from starch and xylan at titers of 460 and 130 mg/L, respectively [16]. Likewise, a recombinant strain of *S. lividans* produced CA at a titer of 490 mg/L from cello-oligosaccharide in complex medium [22].

In this report, a combinatorial study was performed with the purpose of exploring the capacity of various *E. coli* phenoptypes for producing CA and pHCA with different carbon sources, including sugar mixtures similar to lignocellulosic hydrolysates. This study explores novel phenotype/carbon source combinations not previously reported. Isogenic strains employed in this study included W3110 having an active PTS and derived strain VH33 that lacks PTS activity and overexpresses *galP*, thus displaying a PTS^−^ glucose^+^ phenotype [23]. These strains were engineered by the introduction of plasmids bearing genes encoding two different PAL/TAL enzymes as well as *aroG*^fbr^ and *tktA* genes encoding a feedback inhibition resistant version of 3-deoxy-D-*arabino*-heptulosonate 7-phosphate synthase and transketolase, respectively. Characterization of the resulting strains in minimal medium supplemented with single or multiple carbon sources enabled the identification of the phenotype/culture medium combination leading to best CA and pHCA production parameters.

## Materials and methods

### Strains and plasmids

The strains used in this work are described in Table 1. Strain W3110 is a derivative of *E. coli* K-12 [24]. A derivative of W3110 with inactive gene *pheA* was constructed by P1 transduction employing as a donor a mutant from the Keio collection [25]. Mutant selection was performed in Luria broth (LB) with Km 10 μg/mL and by PCR employing primers pheAFwdC GACGCCACAATCAATACACC and pheARevC CGTCGAGCACGATCAGAATA. Plasmid pJLB*aroG*^fbr^*tktA* carries the *aroG*^fbr^ and *tktA* genes encoding a feedback inhibition resistant version of 3-deoxy-D-*arabino*-heptulosonate 7-phosphate synthase and transketolase, respectively [26].

**Table 1:** ***E. coli*** **strains and plasmids used in this study**

**Strain**	**Characteristics**	**Reference**
W3110	*Escherichia coli* F^−^, λ^−^, INV(rrnD^−^ rrnE)1	ATCC 27325
WPJ	W3110 transformed with plasmid pJLB*aroG* ^*fbr*^ *tktA*	This work
WPJRg	W3110 transformed with plasmid pJLB*aroG* ^*fbr*^ *tktA* and pTrcPALRg	This work
WPJAt	W3110 transformed with plasmid pJLB*aroG* ^*fbr*^ *tktA* and pTrcPALAt	This work
W(pheA^−^)Rg	W3110 (*pheA*::Km) transformed with plasmid pJLB*aroG* ^*fbr*^ *tktA* and pTrcPALRg	This work
W(pheA^−^)At	W3110 (*pheA*::Km) transformed with plasmid pJLB*aroG* ^*fbr*^ *tktA* and pTrcPALAt	This work
VH33	Derivative of W3110 (*ΔptsH, ptsI, crr::*Km, *ΔlacI, lacZ::loxP,* P*galP*::P*trc*)	[23]
VPJ	VH33 transformed with plasmid pJLB*aroG* ^*fbr*^ *tktA*	This work
VPJRg	VH33 transformed with plasmid pJLB*aroG* ^*fbr*^ *tktA* and pTrcPALRg	This work
VPJAt	VH33 transformed with plasmid pJLB*aroG* ^*fbr*^ *tktA* and pTrcPALAt	This work
**Plasmids**		
pJLB*aroG* ^*fbr*^ *tktA*	*aroG* ^*fbr*^ under control of the *lacUV5* promoter; and *tktA* under its native promoter, carries *lacI*q and *tet* genes	[26]
pTrc99A	Cloning vector, carries *bla* and *lacI*q genes and *trc* promoter	[27]
pTrcPALRg	PAL gene from *R. glutinis* gene cloned in pTrc99A	This work
pTrcPALAt	PAL gene from *A. thaliana* cloned in pTrc99A	This work

### Cloning of gene PALRg encoding PAL/TAL from R. glutinis

Total RNA was extracted from a culture of *R. glutinis* (ATCC 10788) in complex medium supplemented with 1% L-phenylalanine (L-Phe). RNA was extracted following the TES hot phenol method. Residual DNA was eliminated by treatment with DNAse (DNA-free kit, Ambion). Synthesis of single strand cDNA was performed employing reverse transcriptase M-MuLV with 5 μg of total RNA and primer PALKpnRv (5′AGAGGGTACCCCAAGAAGCGAGTCCTAAGAG) 3′ (RevertAid™ H Minus First Strand cDNA Synthesis Kit Cat. No. K1632, Fermentas Life Sciences). Double strand cDNA was synthesized from 10 ng of single stranded cDNA employing primers PALEcoFw (5′ATAGTAGAATTCCACAGGAAACAGACCATGGCACCCTCGCTCGACTCGA) and PALKpnRv with Long PCR EnzymeMix (ThermoScientific Cat. No.K0182). The PCR product was ligated to plasmid pCR®2.1-TOPO® (Invitrogen TOPO-TA Cloning) and ligation mixture electroporated to electrocompetent *E. coli* DH5 alfa^TM^-t1^R^. The DNA sequence of gen PALRg was verified and then it was subcloned into the expression vector pTrc99A employing sites *EcoR*I and *Kpn*I to generate plasmid pTrcPALRg [27].

### Cloning of gene PALAt encoding PAL from A. thaliana

Total RNA was isolated from *A. thaliana* aerial tissue following a modification of a method reported by Logemann et al. [28]. Residual DNA was eliminated by treatment with DNAse (DNA-free kit, Ambion). First-strand synthesis was performed employing 2 μg of total RNA with reverse transcriptase from SuperScript III (Invitrogen). For second-strand synthesis of gene PALAt, the high fidelity polymerase from Expand High Fidelity (Roche) and primers pal2-5b (5′ AAC CCA TGG ATC AAA TCG AAG CAA TG 3′) and Atpal 2–3 (5′GTT CTA GAG GAA TGC TCT CTT AGC A 3′) were employed. The PCR product was ligated to plasmid pCR®2.1-TOPO® (Invitrogen TOPO-TA Cloning) and ligation mixture electroporated to electrocompetent *E. coli* DH5 alfa™-t1^R^. The DNA sequence of gen PALAt was verified and then it was subcloned into expression vector pTrc99A employing sites *Nco*I and *Xba*I to generate plasmid pTrcPALAt.

### Culture media

Liquid and solid media used during mutant strain construction and inoculum preparation was Luria-Bertani (LB) with antibiotic Km 10 μg/mL, tetracycline (Tc) 30 μg/mL and carbenicilin (Cb) 100 μg/mL when required for mutant strain or plasmid selection. Media employed for strains characterization was M9: Na_2_HPO_4_ 42.26 mM; KH_2_PO_4_ 22.04 mM; NaCl 8.55 mM; NH_4_Cl 18.69 mM; MgSO_4_, 2 mM; CaCl_2_, 0.1 mM; thiamine 29.68 μM, and glucose (55.5 mM), xylose (66.6 mM) or arabinose (66.6 mM). Medium employed to simulate agave lignocellulosic hydrolysate was based on M9 with additional NaCH_3_COO 6.09 mM, and supplemented with 10 g/L of total sugars mixture: glucose (6.66 mM); xylose (58.28 mM); arabinose (5.33 mM) [29]. Medium for cultures with the *pheA*^*−*^ mutant derivative contained 3.02 μM of L-Phe and Km 10 μg/mL. Experiments for determining the toxic effect of various concentrations of CA and pHCA were performed in shake flask cultures in M9 medium supplemented with glucose 55.5 mM.

### Culture conditions

Experiments for determining aromatics acids production were started with an aliquot of an overnight culture in LB medium, inoculating 125 mL shake flasks with 25 mL of M9 medium at a starting OD_600_ of 0.1. The culture was incubated in a horizontal shaking incubator at 37°C, 300 rpm for 10 h. Cells were harvested and used to seed at a starting OD_600_ of 0.1, 250 mL baffled shake flasks containing 50 mL of M9 mineral medium supplemented with 10 g/L of a carbon source. Shake flasks were incubated at 37°C with 300 rpm shaking. All experiments were performed at least in triplicate.

### Analytical methods

Cell concentration was measured as optical density at 600 nm (OD_600nm_) with Spectrophotometer Beckman DU-70. Metabolite concentrations were determined with an HPLC system (600E quaternary bomb, 717 automatic injector, 2410 refraction index, Waters, Milford, MA). For determination of glucose (Glu), xylose (Xyl), arabinose (Ara) and acetic acid, an Aminex HPX-87H column (Bio-Rad, Hercules, CA) was used. Running conditions were: mobile phase, 5 mM H_2_SO_4_; flow, 0.5 ml/min at 50°C. Under these conditions sugars and acetic acid were detected by refraction index. For analysis of L-Phe, L-Tyr, CA and pHCA, HPLC was performed using an Agilent 1100 System (Agilent technologies, Palo Alto, CA) with photodiode array detection. The column used was a Phenomenex Synergi Hydro RP C18, 150 × 4.6 mm, 4 μm (Phenomenex, CA, USA). Running conditions were: mobile phase B, 0.1% TFA in methanol; mobile phase A, 0.1% TFA in water. Flow was set to 1 mL/min and column temperature of 45°C. Mobile phase gradient started with 5% solvent B increasing at 80% in 8 min; this ratio was maintained for 2 min and returned to 5% solvent B in 2 min. From 10 to 12 min reduce solvent B at 5%. Samples were centrifuged and supernatants were filtered with nylon syringe filters

### Kinetic and stoichiometric parameters calculations

Dry cell weight (DCW) concentration was determined considering that 1 OD_600nm_ is equivalent to 0.37 g_DCW_/L [30]. The specific growth rate (*μ*) was calculated by an exponential regression of DCW vs time. The biomass yield from substrate (Y_X/S_) was determined by linear regression of DCW vs substrate consumption at the point of maximum DCW. The specific substrate uptake rate (*q*_*S*_) was calculated by equation *q*_*S*_ = *μ*/Y_X/S_. The product yield from substrate (Y_P/S_) was determined at the point of maximum product concentration. Kinetic characterization of the production strains showed distinct *μ* and aromatic acids production rates; this is caused by differences between PTS^+^ and PTS^−^ glucose^+^ phenotypes, expression of two different PAL/TAL enzymes and growth on three different carbon sources. Therefore, the specific production rate (*q*_*P*_) was calculated using the Luedeking-Piret equation [31], which considers production during both exponential and stationary phases. Kinetic and stoichiometric parameters shown in Additional file 2 were calculated at 36 h.

## Results

The aims of this work were to study the effects on CA and pHCA production of expressing in *E. coli* the genes encoding enzymes having PAL/TAL activities from *A. thaliana* and *R. glutinis*, as well as an assessment on the effects on strain production performance of the PTS^+^ (W3110) and PTS^−^ glucose^+^ (VH33) phenotypes when utilizing various carbon sources, including mixtures in a simulated lignocellulosic hydrolysate (SH). To generate *E. coli* strains with the capacity to synthesize CA and pHCA from a simple carbon source, the first step was to increase carbon flow from central metabolism to the common aromatic pathway, thus enhancing the capacity for the synthesis of L-Phe and L-Tyr, the direct precursors of CA and pHCA. This modification was implemented by transforming *E. coli* W3110 and VH33 with plasmid pJLB*aroG*^fbr^*tktA*, resulting in strains WPJ and VPJ, respectively. These strains were grown in shake flask cultures with minimal medium supplemented with 55.5 mM glucose, 66.6 mM xylose or 66.6 mM arabinose. As expected, accumulation of L-Phe and L-Tyr was detected in the medium (Additional file 1: Figure S1). The final extracellular concentration of these amino acids differed among cultures with the various carbon sources employed, with the highest level detected in culture medium supplemented with arabinose. It should be noted that no L-Phe or L-Tyr were detected in the supernatant of cultures with strains W3110 or VH33.

The conversion of L-Phe and L-Tyr to CA and pHCA is dependent on PAL and TAL activities, respectively. *E. coli* lacks these enzymes, therefore, genes PALRg and PALAt encoding enzymes with PAL/TAL activities from *R. glutinis* and *A. thaliana*, respectively, were cloned in an expression vector to generate plasmids pTrcPALRg and pTrcPALAt. To generate CA and pHCA production strains, *E. coli* WPJ and VPJ were transformed with pTrcPALRg or pTrcPALAt, resulting in strains WPJRg, WPJAt, VPJRg and VPJAt.

### Production of CA and pHCA in cultures with minimal medium supplemented with glucose, xylose or arabinose as carbon source

Shake flask cultures with strains WPJRg, WPJAt, VPJRg and VPJAt were performed in minimal salts medium supplemented with glucose, xylose or arabinose. For cultures with glucose as the carbon source, it can be observed that pHCA was produced only by strains WPJRg and VPJRg that express *R. glutinis* PAL/TAL. Strain VPJRg produced 107 μM pHCA with an Y_pHCA/Glc_ of 1.03 μmol_pHCA_/mmolC; these values are 20 fold higher when compared to results from strain WPJRg (Additional file 2: Table S1, Additional file 1: Figure S2). The compound CA was produced by the four characterized strains, however, the higher titers were observed in cultures with strains WPJAt and VPJAt (~530 μM). Although both strains displayed similar titer values, the yield from glucose (Y_CA/Glu_) and the specific rate of production observed for strain VPJAt were 3.6 and 1.5 fold higher when compared to strain WPJAt.

Strain performance for production of CA and pHCA was also evaluated with xylose and arabinose as carbon sources. These sugars are internalized by non-PTS transport proteins and are metabolized via the pentose phosphate pathway (Figure 1, Additional file 2: Tables S1, S2 and S3). When grown with xylose, strains WPJRg and VPJRg produced pHCA titers of 25 and 15 μM, respectively (Additional file 1: Figure S3). The pHCA titer produced by strain WPJRg from xylose was 5 fold higher when compared to cultures with glucose. In contrast, a 6.9 fold reduction in pHCA titer was observed for strain VPJRg when comparing cultures with xylose to cultures with glucose. Production of CA was improved in strains derived from W3110 when comparing xylose to glucose as a carbon source, with strain WPJAt displaying the highest observed titer (736.8 μmol/L), corresponding to a 1.4 fold improvement over glucose.

In cultures with arabinose as sole carbon source, strain WPJRg displayed a pHCA titer of 76.7 μM with an Y_pHCA/Ara_ of 0.437 μmolpHCA/mmolC. These values are 8.8 and 15.4 fold higher when compared to cultures with glucose. In these cultures, CA was produced by all strains, however, as it was observed in cultures with other sugars, the higher titers were produced by strains expressing PAL from *A. thaliana.* Strain WPJAt displayed an Y_CA/Ara_ 1.6 fold higher when compared to results with glucose and a *q*_*CA-Ara*_ of 368.9 μmolCA/g_DCW_hr. The CA titer produced by WPJAt was 1022 μM, the highest observed in this study (Additional file 2: Table S3, Additional file 1: Figure S4).

### Production of CA and pHCA in cultures with minimal medium supplemented with a SH

Lignocellulose hydrolysates are a source of fermentable sugars containing mainly pentoses, hexoses and acetate. To determine CA and pHCA production capacity on medium containing a mixture of carbon sources similar to that obtained from a hydrolysate, cultures were performed in minimal medium supplemented with sugars and acetate in a similar proportion as that found after thermochemical hydrolysis of agave bagasse [29]. The SH contained xylose (53.28 mM), glucose (6.66 mM), arabinose (5.33) and acetic acid (6.09 mM). Results from cultures of the four strains with SH showed similarity to cultures with xylose as a sole carbon source. This is expected considering that xylose is the main component in the SH sugar mixture. However, some differences were detected when comparing data from cultures in the SH to those from cultures with xylose. Strains WPJRg and VPJRg, displayed a 26 and 42% increase in Y_pHCA/SH_, respectively, when compared to results of cultures with xylose. In addition, a 12 and 44% increase in *q*_*pHCA-SH*_ was detected when performing the same comparison. Cultures with strain VPJAt displayed a similar CA titer and a 30% increase in *q*_*CA-SH*_ when compared to cultures with xylose (Additional file 2: Table S4).

In these cultures, differences in the pattern of carbon source utilization are evident (Additional file 1: Figure S5). It can be observed in the culture with strain WPJRg that glucose exerts total and partial repression in the consumption of xylose and arabinose, respectively. In contrast, in the culture with VPJRg, the simultaneous utilization of the three sugars is evident. It should be noted that acetate in the SH was not consumed by any of the four strains, on the contrary, this organic acid was produced and its concentration increased in the culture medium.

### Effects on CA and pHCA synthesis of inactivating gene ***pheA*** from the L-Phe biosynthetic pathway

The above results showed, as it has been previously reported, that PAL/TAL from *R. glutinis* can employ L-Phe or L-Tyr as substrates and thus produce both CA and pHCA [5]. In the strains that expressed PAL/TAL from *R. glutinis*, the amount of produced pHCA was always lower than CA. To improve the pHCA/CA ratio, a strategy was followed to reduce availability of substrate L-Phe by inactivating gene *pheA*, encoding enzyme CM-PDT (Figure 1). This approach was tested on strain W3110 since it displayed the best pHCA production parameters with SH. Gene *pheA* was inactivated in W3110 and resulting mutant was transformed with plasmids PJLB*aroG*^*fbr*^*tktA* and pTrcPALRg or pTrcPALAt to generate strains W(pheA^−^)Rg and W(pheA^−^)At (Table 1). These strains were characterized in shake flask cultures with a SH as carbon source (Additional file 2: Table S4, Additional file 1: Figure S6). Strain W(pheA^−^)Rg displayed an 8-fold increase in Y_pHCA/SH_ as well as a 2.9 fold increase in *q*_*pHCA-SH*_ when compared to values from strain WPJRg when growing in SH. At 12 h of culture time, a titer of approximately 322 μM pHCA was produced by W(pheA^−^)Rg, this represents a near 10-fold increase over the amount of pHCA produced by WPJRg (39 μM). It is noteworthy that the CA titer was not lower for W(pheA^−^)Rg when compared to cultures with strain WPJRg, however, the pHCA/CA ratio increased from 0.93 for WPJRg to 5.1 for strain W(pheA^−^)Rg. When compared to results from W(pheA^−^)At cultures, the Y_CA/SH_ and *q*_*CA*_ of WPJAt were 22.8 and 49.3% lower.

## Discussion

The current trends in the development of biotechnological production processes for chemical compounds follows the use of minimal media supplemented with sustainable raw materials, such as lignocellulosic hydrolysates. This type of raw material contains a mixture of pentoses and hexoses; mainly xylose, arabinose and glucose. One of the mayor challenges in developing a biotechnological process based on the use of lignocellulosic hydrolysates is the isolation or development of production organisms capable of efficiently utilizing all the different sugars in the mixture. Most studies related to microbial CA and pHCA production are based on the use of culture media containing glucose as raw material and in some cases also tryptone, peptone or other complex nutrients [3,5,6,14-16]. However, there are some reports where xylose, raffinose, glycerol, cellobiose, avicel and cellulose are also employed as carbon sources [22,32]. The bacterium *E. coli* has the natural capacity for consuming the main sugar components in a typical lignocellulosic hydrolysate. For this reason, in this study, *E. coli* W3110 was chosen as the parent host for developing CA and pHCA production strains. An isogenic derivative of W3110 having a PTS^−^ glucose^+^ phenotype (VH33) was also employed, since it has been demonstrated that PTS inactivation can lead to improved capacity for consuming sugar mixtures [33]. The capacity for synthesizing the aromatic acids CA and pHCA was engineered in these strains by transforming them with plasmids carrying genes encoding for enzymes with PAL/TAL activities. The PAL/TAL enzyme from *R. glutinis* has been shown to have a higher affinity for L-Tyr than L-Phe [15,18]. Whereas the PAL1 isozyme from *A. thaliana*, has been expressed in *E. coli* for the synthesis of CA as a precursor in heterologous pathways for the production of flavonoids or styrene [17,34].

**Figure 2:**
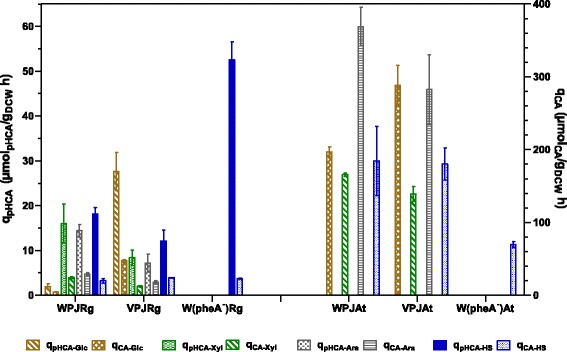
**Specific rates of production of CA and pHCA by strains grown in glucose, xylose, arabinose or simulated hydrolysate (SH).**

**Figure 3:**
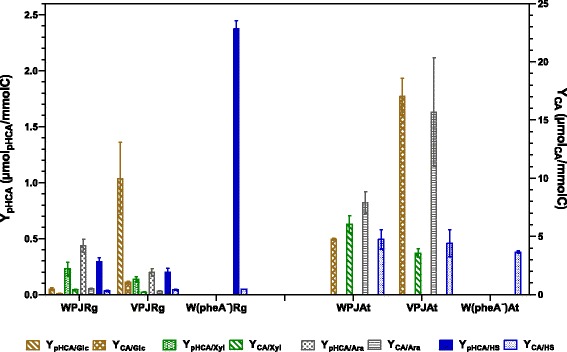
**Yields of CA and pHCA from carbon sources by strains grown in glucose, xylose, arabinose or simulated hydrolysate (SH).**

In cultures employing glucose as the carbon source, strains WPJRg and VPJRg produced both CA and pHCA, whereas strains WPJAt and VPJAt produced only CA (Figures 2 and 3). This is the result of the distinct affinities for substrates displayed by the two expressed PAL/TAL enzymes, as it will be discussed below. Under these growth conditions, a clear improvement in specific productivity and yield from glucose for CA and pHCA was observed when comparing strains with the PTS^−^ glucose^+^ and PTS^+^ phenotypes. The positive effect of PTS inactivation results from avoiding phosphoenolpyruvate consumption during glucose import. In strain VH33, PTS activity is replaced by glucose transport dependent on the GalP glucose/galactose symporter and ATP-dependent phosphorylation by glucokinase. The improved performance on aromatics production when employing glucose as carbon source has been reported for other PTS^−^ glucose^+^ strains and the particular level of improvement is dependent on specific product and culture conditions [13,26].

In cultures employing xylose or arabinose as carbon sources, strain WPJRg displayed higher specific productivity and Y_pHCA/Substrate_ values when compared to VPJRg. When strain WPJRg was cultured with arabinose, it displayed a 3 fold higher pHCA titer and 2 fold higher Y_pHCA/Ara_ when compared to results with xylose as carbon source. However, when growing with xylose, strain WPJAt displayed Y_CA/Xyl_ and a CA titer about 2-fold higher than that observed for VPJAt (Figures 2 and 3). When growing on arabinose, strain WPJAt produced the highest observed CA titer of all studied strains and growth conditions (1022 μM). This is in part explained by the higher biomass concentration of WPJAt when compared to VPJAt. This is the result of a *q*_*Ara*_ which is 30% higher when compared to VPJAt. These data show a clear aromatics acids production performance advantage with xylose or arabinose of a strain with a functional PTS when compared to a mutant having a PTS^−^ glucose^+^ phenotype. The internalization of xylose or arabinose is not dependent on PTS activity, therefore, no difference in production capacity was expected when comparing strains with the PTS^+^ or the PTS^−^ glucose^+^ phenotypes. A possible explanation for these results can be provided considering that inactivation of PTS causes an impairment on the capacity to synthesize cAMP, a molecule that is required together with the global regulator CRP, for inducing expression of the operons encoding transporters and enzymes involved in xylose and arabinose catabolism [35,36].

Cultures performed with single carbon sources enabled an overview and a comparison of production performance for CA and pHCA among isogenic strains with PTS^+^ or PTS^−^glucose^+^ phenotypes. However, the biotechnological sustainable production of these and other chemicals would preferably be dependent on the use of lignocellulosic hydrolysates as raw material. To determine the production parameters for CA and pHCA with a culture medium containing sugars frequently found lignocellulosic hydrolysate, a culture medium was formulated containing a mixture of xylose, arabinose, glucose and acetate. Results from experiments under these conditions showed a similar production performance for CA and pHCA among the PTS^+^ or the PTS^−^ glucose^+^ phenotypes. When considering the pattern of sugar consumption, catabolic repression by glucose was observed in cultures with WPJAt. In contrast, simultaneous consumption of carbon sources was evident in strain VPJAt (PTS^−^ glucose^+^). This response has been previously reported for other PTS^−^ glucose^+^ strains growing with two or three different sugars [33]. However, even though strains WPJAt and WPJRg displayed sequential consumption of sugars in the SH, they showed slightly better production parameters when compared to PTS^−^ glucose^+^ strains (Figures 2 and 3). This can be explained by the relatively high proportion of xylose in the SH sugar mixture. When considering results of experiments with single sugars, it is clear that PTS^−^ glucose^+^ strains could be advantageous when employing hydrolysates from lignocellulosic sources that yield a higher proportion of glucose when compared to the SH employed here.

The experiments performed here with *E. coli* strains expressing PAL/TAL from *A. thaliana* and *R. glutinis* showed that only the latter deaminated L-Tyr to produce pHCA. The PAL/TAL from *R. glutinis* could also employ L-Phe as substrate, producing CA at a titer at least 2 fold higher than pHCA. It has been reported that PALRg has higher affinity towards L-Tyr, however the *k*_*cat*_/K_m_ for L-Phe (1.9 × 10^4^ mM^−1^ s^−1^) is higher than for L-Tyr (0.52 × 10^3^ mM^−1^ s^−1^) [36]; it should also be noted that strains WPJ and VPJ produced at least two fold more L-Phe than L-Tyr with most carbon sources employed here (Additional file 1: Figure S1). These data explain why strains expressing *R. glutinis* PAL/TAL produce more CA than pHCA. The enzyme from *A. thaliana* expressed in strains WPJAt and VPJAt generated the highest levels of CA under all studied conditions. Comparison of kinetic parameters among strains and different culture conditions showed lower μ and final biomass concentrations in strains expressing PAL/TAL from *A. thaliana* when compared to expression of PAL/TAL from *R. glutinis*. This could be explained considering that PAL from *A. thaliana* is consuming intracellular L-Phe as a substrate for CA production, therefore limiting this amino acid for growth requirements. In agreement with this hypothesis, the *k*_*cat*_/K_m_ for L-Phe is higher for the enzyme from *A. thaliana* (5.12 × 10^7^ mM^−1^ s^−1^) [19] when compared to *R. glutinis* (1.9 × 10^4^ mM^−1^ s^−1^) [37].

To increase production of pHCA in a strain expressing PALRg, a strategy was followed wherein L-Phe availability would be reduced. This strategy was implemented by inactivating in strain W3110 the gene *pheA* that encodes enzyme CM-PDH from the L-Phe biosynthetic pathway. Cultures with strain W(*pheA-*)At showed that this strain still produced CA, but with a 2.6-fold lower *q*_*CA-HS*_ when compared to strain WPJAt. This result can be explained considering that conversion of prephenate to phenylpyruvate can proceed in absence of enzyme catalysis [38]. Therefore, it can be assumed that consumption of L-Phe by PAL from *A. thaliana* drives flux from chorismate to CA in the absence of CM-PDT activity. The inactivation of *pheA* caused an increase in the pHCA/CA ratio produced by strain W(pheA^−^)Rg (5.15) when compared to WPJRg (0.93). It was also observed that the Y_pHCA/HS_ and *q*_*pHCA*_ improved as a consequence of *pheA* inactivation. Similar results have been reported for a *Pseudomonas putida* strain that was subjected to random mutagenesis to increase L-Phe production and again randomly mutagenized to generate an L-Phe auxotroph [6]. However, the mutations causing such phenotype have not been determined.

Both CA and pHCA are toxic compounds for *E. coli* and other microorganisms [3,39]. To determine the toxic effect of these two aromatic acids under our experimental conditions, cultures were performed in M9 medium with glucose supplemented with various amounts of CA and pHCA. When compared to cultures in M9 medium, a 10 and 6% reduction in growth rate was observed in M9 with 10 mM pHCA or CA, respectively (Additional file 1: Figure S7). It should be noted that these concentrations of aromatic acids are at least three orders of magnitude higher than the titers observed in our production experiments. Therefore, it is unlikely that toxic effects of pHCA or CA could have a negative effect on production capacity. However, under growth conditions designed to maximize productivity, such as fed-batch cultures, the titers of these compounds could reach a toxic level. Under such conditions, strategies like the overexpression of efflux proteins or the use of naturally-resistant organisms such as a solvent-tolerant *P. putida* strain, should enable the development of processes for the high-titer production of CA and pHCA [3,6].

The characterization of several *E. coli* strains growing with various carbon sources enabled the identification of particular combinations yielding the best production performances. Product titer is dependent on biomass concentration and this parameter varies among the different carbon sources employed. Therefore, the yield from carbon source and the specific productivity can be considered better parameters for evaluating strains performance. For the case of pHCA, strain W(pheA^−^)Rg displayed the best *q*_*pHCA*_ (52.57 μmol/h*g_CDW_) and Y_pHCA/HS_ values when growing on SH. Whereas for CA, strain WPJAt displayed the highest *q*_*CA*_ (368.9 μmol/h*g_CDW_) and Y_CA_ values with arabinose (Figures 2 and 3). However, when considering carbon source cost and availability, other strain/carbon source combinations could be attractive from a biotechnological standpoint. Considering the diversity regarding strain phenotypes, culture media and growth conditions, it is not feasible to perform a direct comparison on the performance of the strains studied here with other published works. Nevertheless, some reported data will be discussed to provide a framework for our results. Table 2 provides a list of production parameters and culture conditions from publications related to microbial CA and pHCA production, the utilized microorganisms include *E. coli*, *S. cerevisiae*, *P. putida* and *Streptomyces lividans*. A wide variety of genetic modification strategies were applied to generate these production strains. However, the heterologous expression of genes encoding enzymes with PAL/TAL activities was always employed. What follows is a comparison of strain performance among those studies providing data that can be related to our study. Similar or higher CA titers to those observed for *E. coli* strain WPJAt with arabinose have been reported with *S. lividans* expressing the PAL from *Streptomyces maritimus* and employing culture medium containing tryptone and carbon sources such as xylose, glycerol or xylooligosacharides [16]. However, these titers were reached after 6 to 8 days of culture time. In contrast, the *E. coli* strain reported here produced similar CA titers in 36 h and employing minimal salts medium. A study employing a strain derived from solvent-tolerant *Pseudomonas putida* S12 reports a *q*_*pHCA*_ of 84 μmol/h*g_CDW_ from glucose [6]. With another strain derived from this bacterium, a *q*_*CA*_ of 60 μmol/h*g_CDW_ from glycerol in a fed-batch process was reported [21]. These data show that *P. putida* S12 is currently the microorganism displaying the best production parameters for CA and pHCA, likely as a consequence of its solvent-tolerant capacity. It remains to be determined if the *E. coli* production strains developed in this study could be improved by increasing their resistance to aromatic acids toxicity by following an strategy based on overexpressing native or heterologous efflux proteins [3].

**Table 2:** **Comparison of microbial CA and pHCA production parameters and culture conditions**

**Organism**	**PAL/TAL**	**Carbon source**	**Culture medium**	**Culture conditions**	**pHCA (μM)**	**Q**_**pHCA**_ **(μmol/Lh)**	**CA (μM)**	**Q**_**CA**_ **(μmol/Lh)**	**Reference**
*E. coli (*W3110)	*R. glutinis*	Glucose	Mineral	30°C 24 h	42.4	1.76	32.9	1.37	[5]
				Baffled Flask					
*E. coli* (DH10B)	*R. glutinis*	Glucose	Mineral	30°C 18 h	280	15.5	280	15.5	[5]
				Baffled Flask					
*E. coli*(ATCC 31884)	*R glutinis*	Glucose	Mineral	30°C 18 h	150	8.33	380	21.1	[5]
				Baffled Flask					
*S. cerevisiae*	*R. glutinis*	Glucose	Mineral	30°C 48 h	14.3	0.29	-	-	[5]
				Baffled Flask					
E. coli (BL21*AI)	*Trichosporon cutaneum*		LB	30°C 16 h	432	27	843	52.68	[16]
*P. putida S12 C1*	*Rhodosporidium. toruloides*	Glucose	Mineral	30°C	224	84 (μmol/h*g_DCW_)	350	-	[6]
				Shake flask					
*P. putida S12 C3*	*R. toruloides*	Glucose	Mineral	30°C	860	84 (μmol/h*g_DCW_)	70	-	[6]
				Shake flask					
*P. putida S12 C3*	*R. toruloides*	Glucose	Mineral	30°C	10,600	24 (μmol/h*g_DCW_)	150	-	[6]
				Fed-batch					
*S. lividans*	*R. sphaeroides*	Glucose	3% TSB	28°C	4755	28.5	-	-	[31]
			5% Triptone	168 h					
*P. putida* S12	*R. toruloides*	Glucose	Mineral	30°C	-	-	415	12	[21]
				Shake flask					
				ON					
*P. putida* S12	*R. toruloides*	Glucose	Mineral	30°C	-	-	5,000	166	[21]
				Fed-batch					
				30 h					
				N_2_ limitation					
*P. putida* S12	*R. toruloides*	Glycerol	Mineral	30°C	-	-	5,400	108 (60 μmol/h*g_DCW_)	[21]
				Fed-batch					
				50 h					
				N_2_ limitation					
*S. lividans*	*S. maritimus*	Glucose	TSB	28°C	-	-	540	5.62	[22]
				Shake flask					
				96 h					
*S. lividans*	*S. maritimus*	Glucose	TSB	28°C	-	-	1417	11.8	[22]
			Triptone	Shake flask					
				120 h					
*S. lividans*	*S. maritimus*	Xylose	TSB	28°C	-	-	2,024	14.05	[22]
			Triptone	Shake flask					
				144 h					
VPJRg	*R. glutinis*	Glucose	M9	16 h	91.45	5.72	196.15	12.26	This work
VPJAt	*A. thaliana*	Glucose	M9	36 h	-	-	529.94	14.72	This work
WPJAt	*A. thaliana*	Arabinose	M9	31 h	-	-	1,022.36	28.4	This work
W(pheA^−^)Rg	*R. glutinis*	SH	M9	16 h	355.87	22.24	105.45	6.59	This work
WPJAt	*A. thaliana*	SH	M9	31 h	-	-	377.87	12.19	This work

By employing PAL/TAL enzymes from two different sources, it was possible to generate *E. coli* strains for the production of CA and pHCA from simple carbon sources in a minimal medium. A comparison of PTS^+^ and PTS^−^ glucose^+^ strains enabled the identification of the specific phenotype showing the best production performance with various carbon sources. This combinatorial study provides useful parameters to be considered for the future design of strains for the production of this type of aromatic compounds. In the case of pHCA production, a challenge is to generate a strain that synthesizes this compound in the absence of CA. Among potential improvements to the pHCA production system, it can be considered the use of a PAL/TAL enzyme that only employs L-Tyr as substrate. So far, an enzyme with such characteristic has not been reported. It remains to be determined if a PAL/TAL enzyme that is selective for L-Tyr could be found in the natural diversity. Alternatively, protein engineering could be employed to increase substrate specificity towards L-Tyr of an existing PAL/TAL enzyme. The strains developed here can be considered as production platforms for the synthesis of compounds derived from CA and pHCA such as p-hydroxystyrene and phenylpropanoids [20,39].

## Additional files

Additional file 1:
**Figure S1.** Production of L-Phe and L-Tyr by strains WPJ and VPJ grown in M9 medium supplemented with glucose, xylose, arabinose or simulated hydrolysate (SH). **Figure S2.** Growth and production profiles of W3110 and VH33 derivatives in shake flask cultures with glucose as carbon source. **Figure S3.** Growth and production profiles of W3110 and VH33 derivatives in shake flask cultures with xylose as carbon source. **Figure S4.** Growth and production profiles of W3110 and VH33 derivatives in shake flask cultures with arabinose as carbon source. **Figure S5.** Growth and production profiles of W3110 and VH33 derivatives in shake flask cultures with a simulated hydrolysate (SH) as carbon source. **Figure S6.** Growth and production profiles of W(pheA^−^)Rg y W(pheA^−^)At in shake flask cultures with a simulated hydrolysate (SH) as carbon source. **Figure S7.** Relative growth rate of *E. coli* W3110 in M9 medium supplemented with glucose and various concentrations of CA and pHCA. Additional file 2:
**Table S1.** Kinetic parameters of growth strains in minimal medium with glucose 55.5 mM (10 g/L). **Table S2.** Kinetic parameters of growth strains in minimal medium with xylose 66.6 mM (10 g/L). **Table S3.** Kinetic parameters of growth strains in minimal medium with arabinose 66.6 mM (10 g/L). **Table S4.** Kinetic parameters of cultures with engineered strains grown in a minimal medium with simulated hydrolysate (SH). Glucose 6.66 mM (1.2 g/L), xylose 53.3 mM (8 g/L), arabinose 5.33 mM (0.8 g/L) and acetate 6.09 mM (0.5 g/L). Total sugars 62.29 mM (10 g/L). Kinetic and stoichiometric parameters shown in supplementary tables were calculated at 36 h.
