# Supplementary material for: Production of cinnamic and p-hydroxycinnamic acid from sugar mixtures with engineered Escherichia coli
Source: Microb Cell Fact. 2015 Jan 16;14:6. doi: 10.1186/s12934-014-0185-1 (PMC4305220; doi:10.1186/s12934-014-0185-1)
Supplement: Additional file 1: — Figure S1. Production of L-Phe and L-Tyr by strains WPJ and VPJ grown in M9 medium supplemented with glucose, xylose, arabinose or simulated hydrolysate (SH). Figure S2. Growth and production profiles of W3110 and VH33 derivatives in shake flask cultures with glucose as carbon source. Figure S3. Growth and production profiles of W3110 and VH33 derivatives in shake flask cultures with xylose as carbon source. Figure S4. Growth and production profiles of W3110 and VH33 derivatives in shake flask cultures with arabinose as carbon source. Figure S5. Growth and production profiles of W3110 and VH33 derivatives in shake flask cultures with a simulated hydrolysate (SH) as carbon source. Figure S6. Growth and production profiles of W(pheA−)Rg y W(pheA−)At in shake flask cultures with a simulated hydrolysate (SH) as carbon source. Figure S7. Relative growth rate of E. coli W3110 in M9 medium supplemented with glucose and various concentrations of CA and pHCA. [file 12934_2014_185_MOESM1_ESM.ppt]

## Slide 1
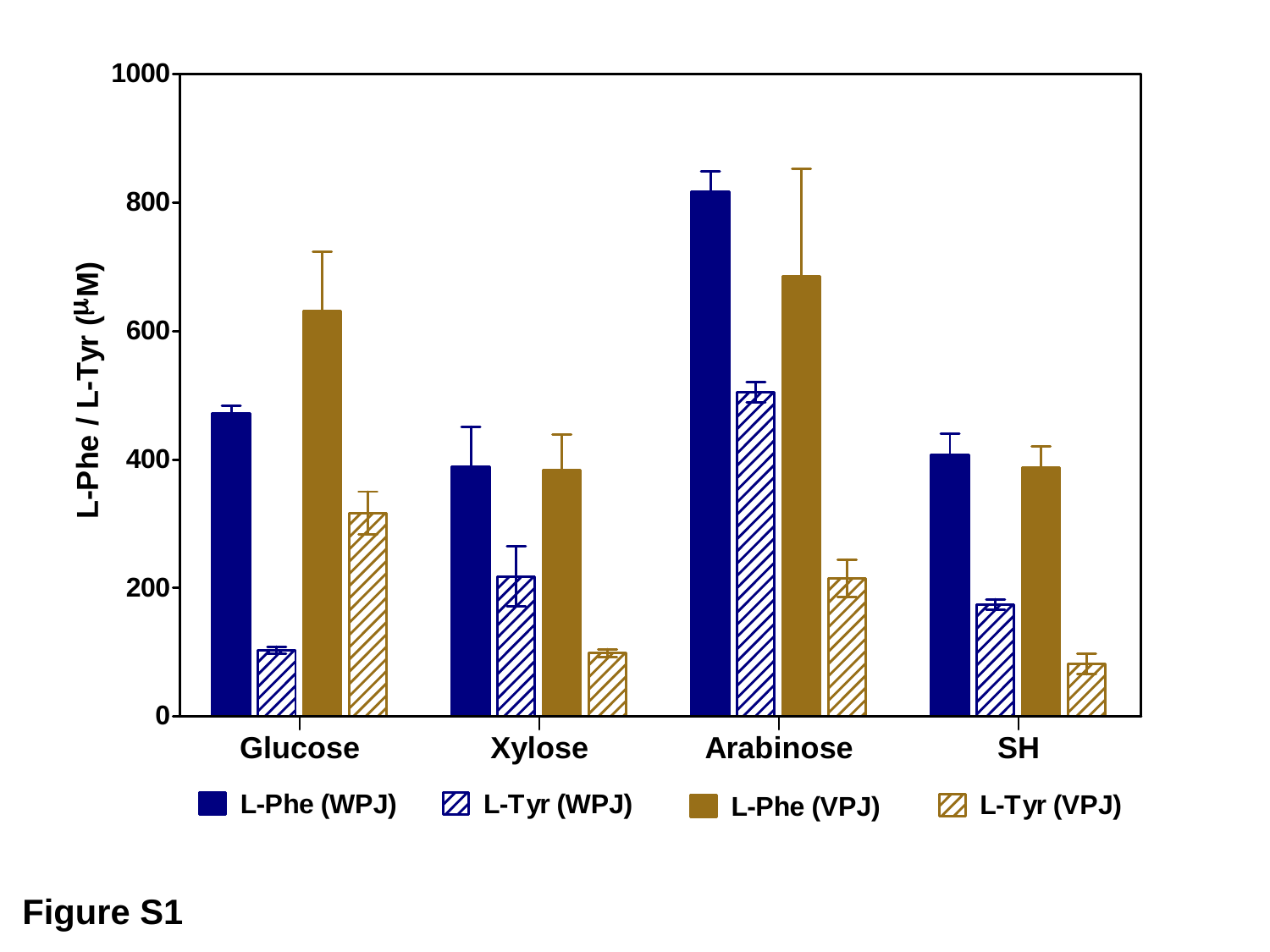

Figure S1

## Slide 2
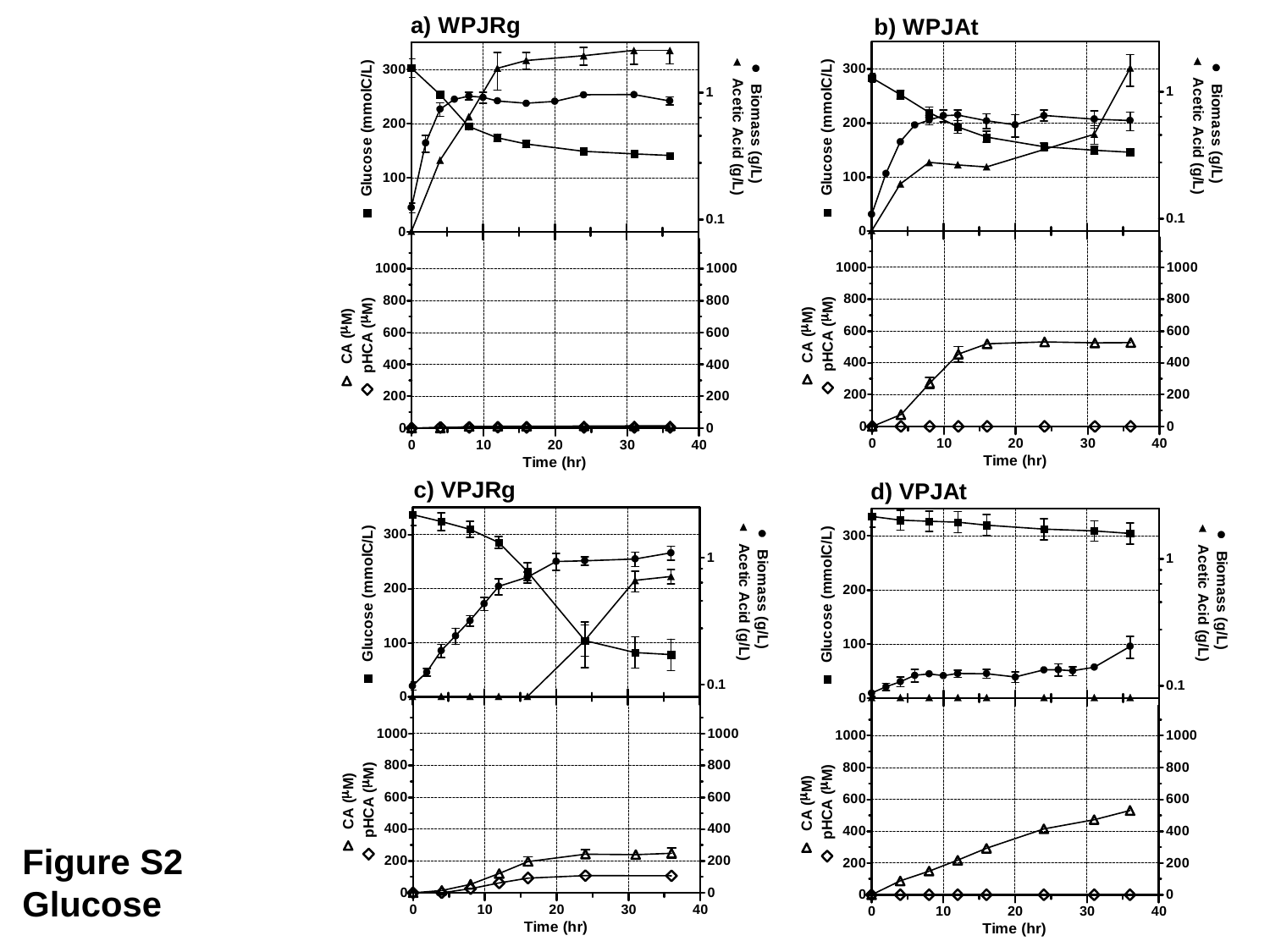

Figure S2
Glucose

## Slide 3
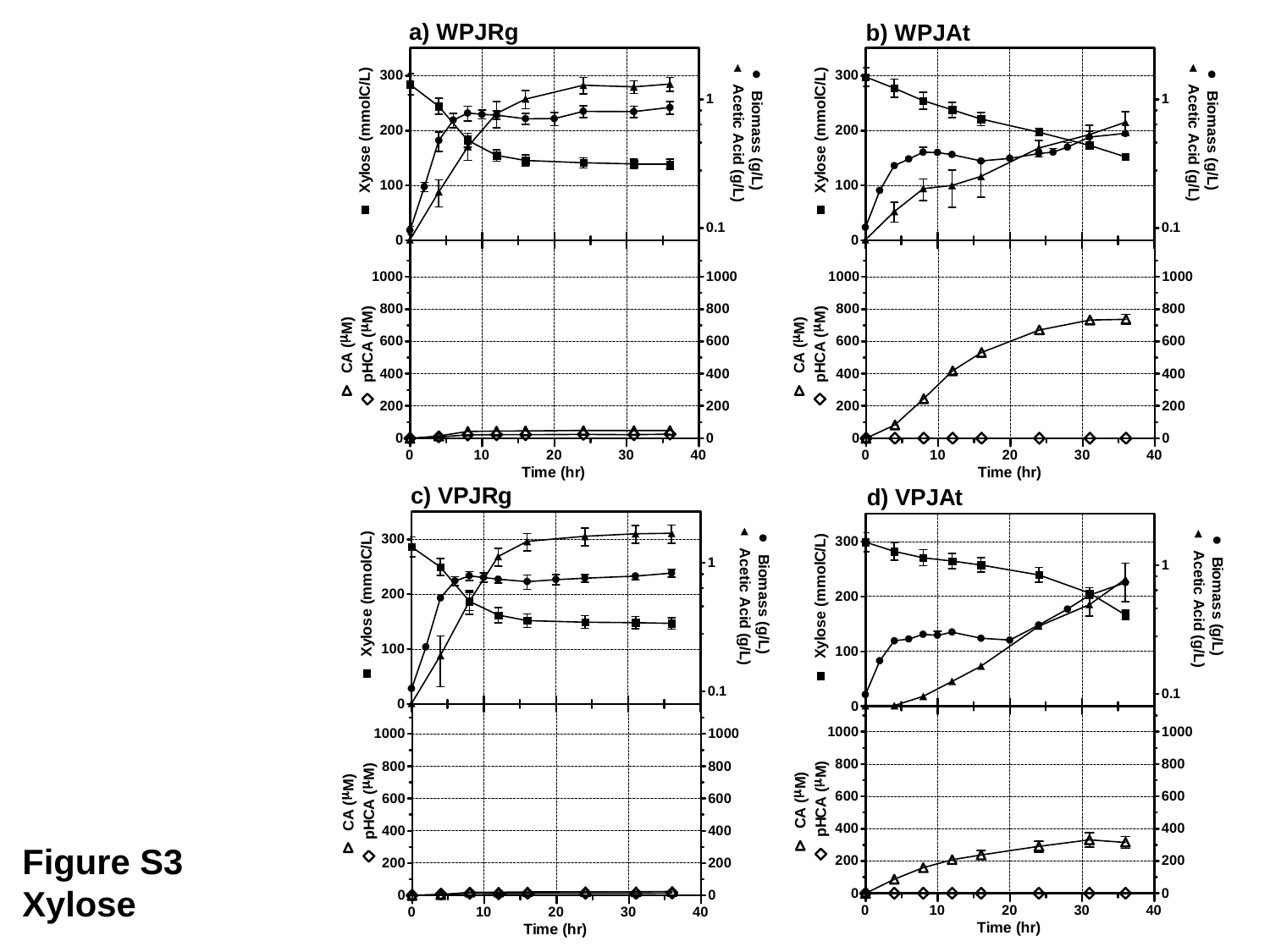

Figure S3
Xylose

## Slide 4
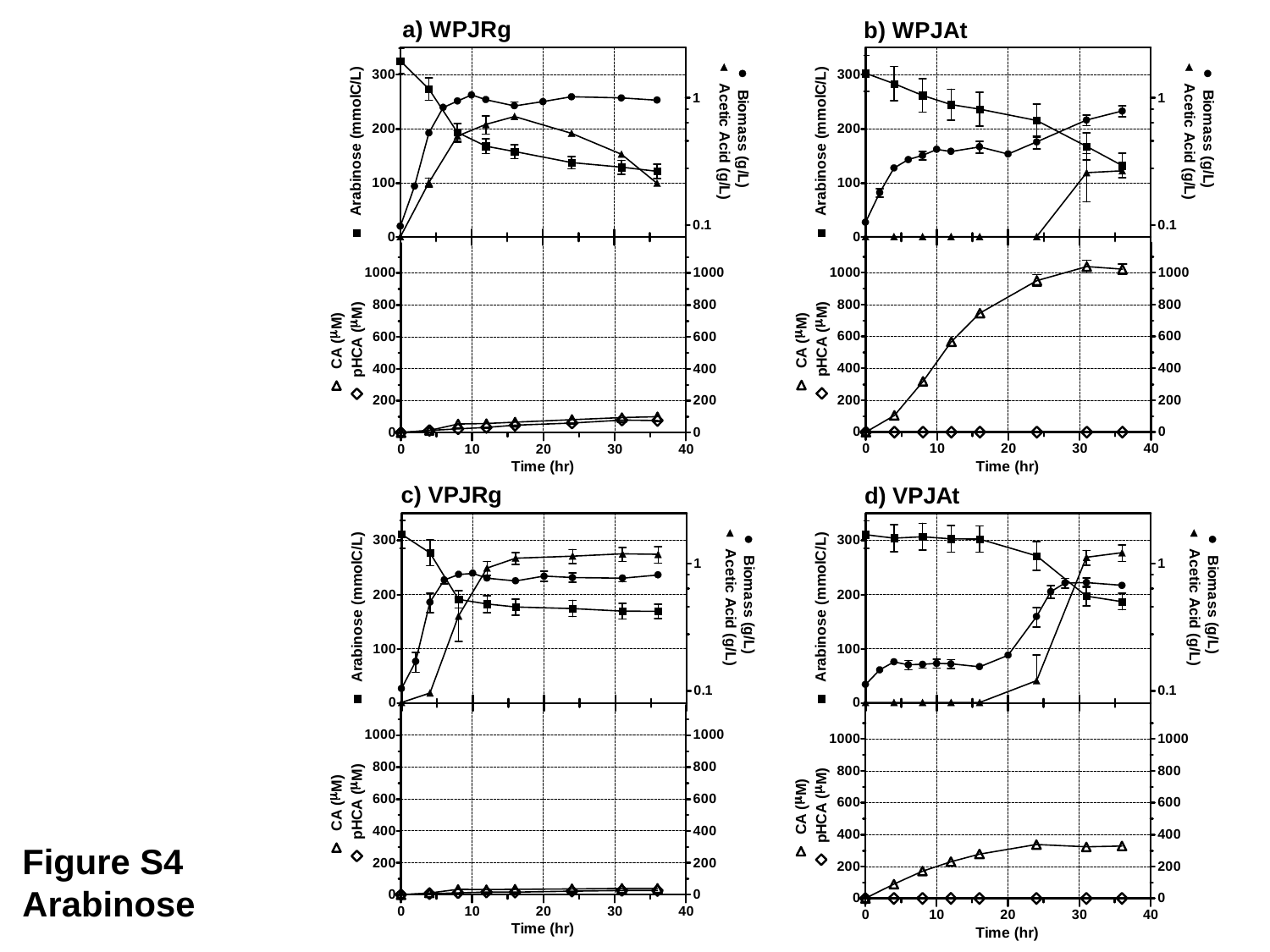

Figure S4
Arabinose

## Slide 5
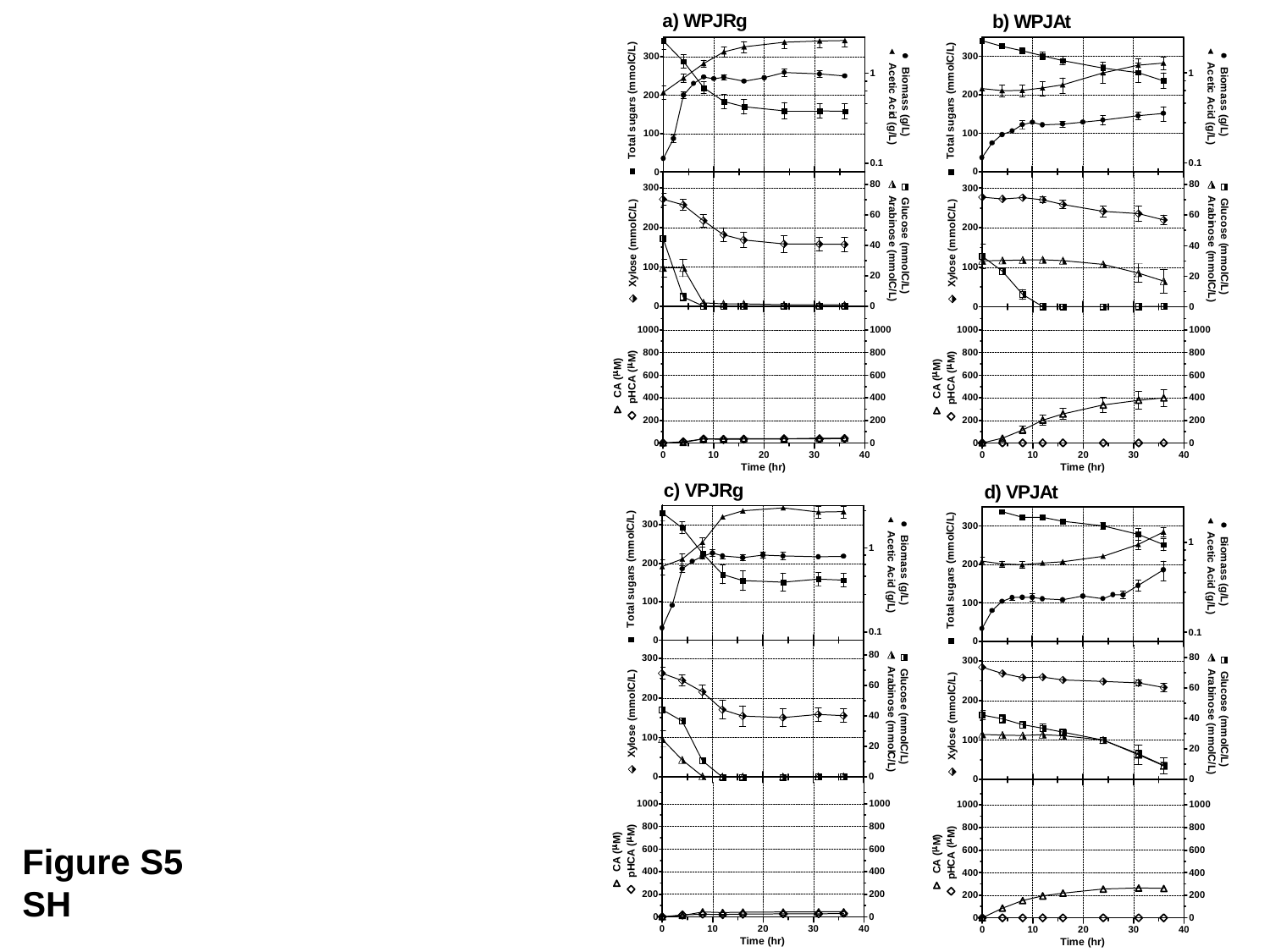

Figure S5
SH

## Slide 6
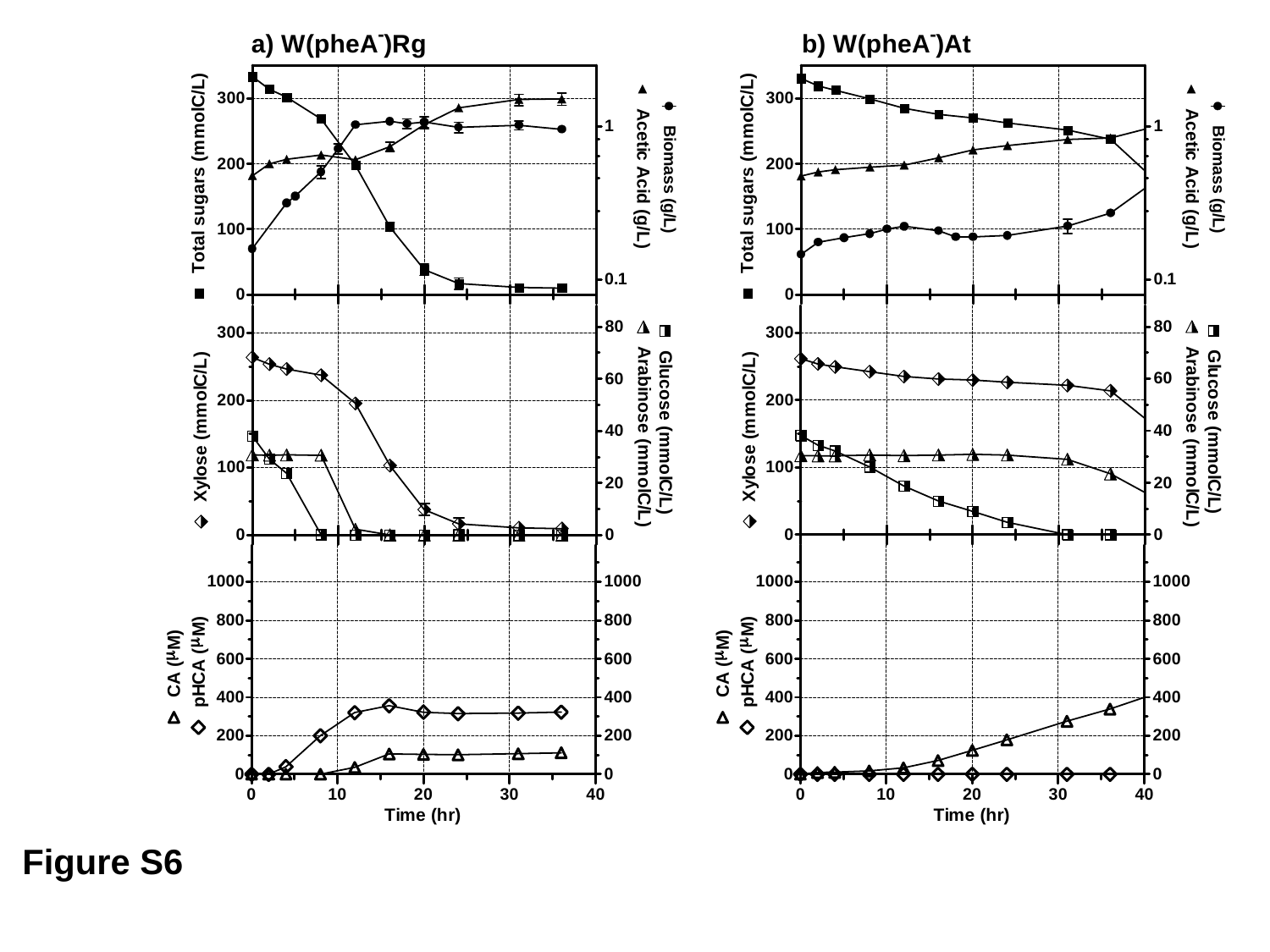

Figure S6

## Slide 7
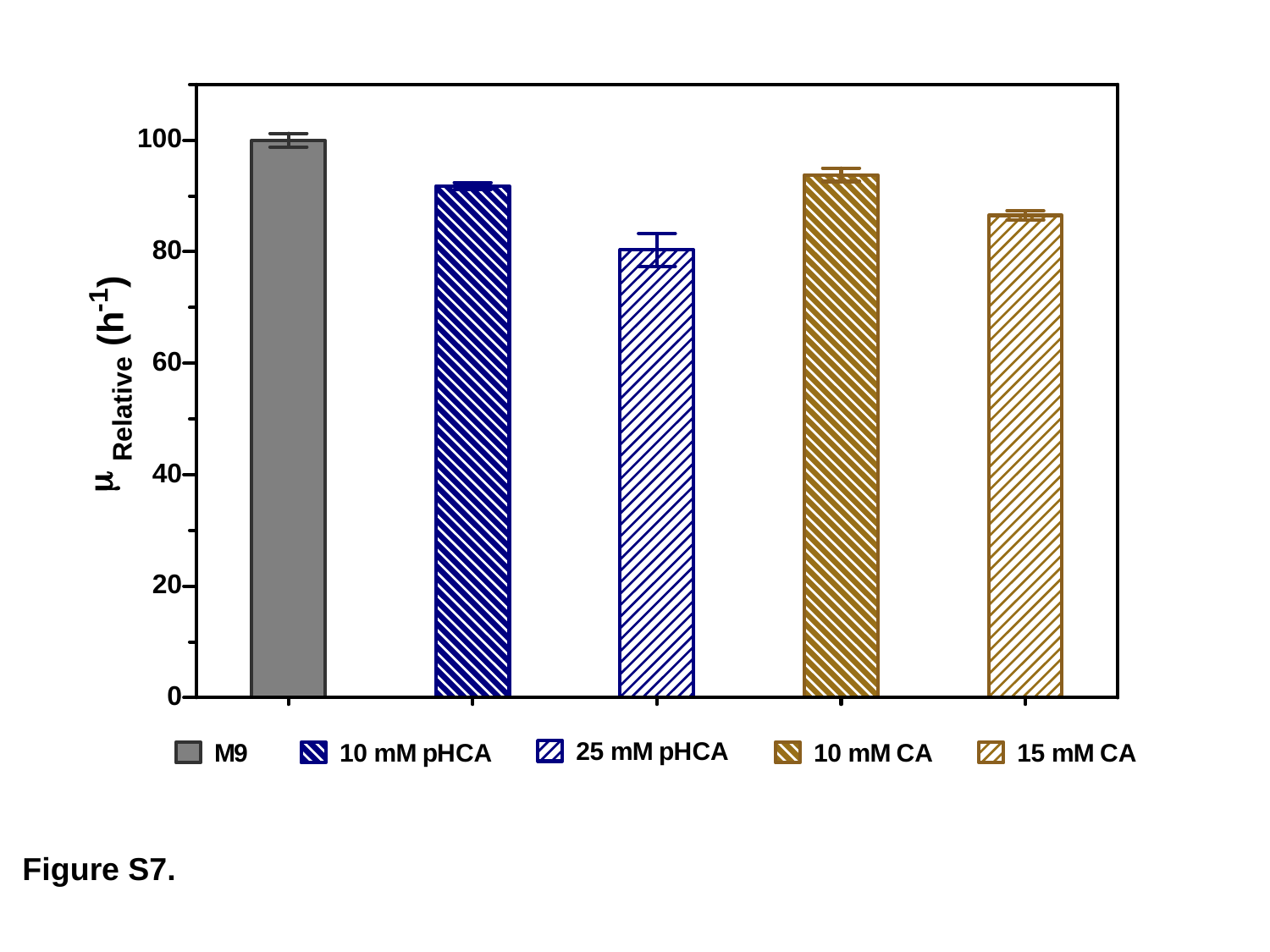

Figure S7.
