# Supplementary material for: Production of cinnamic and p-hydroxycinnamic acid from sugar mixtures with engineered Escherichia coli
Source: Microb Cell Fact. 2015 Jan 16;14:6. doi: 10.1186/s12934-014-0185-1 (PMC4305220; doi:10.1186/s12934-014-0185-1)
Supplement: Additional file 2: — Table S1. Kinetic parameters of growth strains in minimal medium with glucose 55.5 mM (10 g/L). Table S2. Kinetic parameters of growth strains in minimal medium with xylose 66.6 mM (10 g/L). Table S3. Kinetic parameters of growth strains in minimal medium with arabinose 66.6 mM (10 g/L). Table S4. Kinetic parameters of cultures with engineered strains grown in a minimal medium with simulated hydrolysate (SH). Glucose 6.66 mM (1.2 g/L), xylose 53.3 mM (8 g/L), arabinose 5.33 mM (0.8 g/L) and acetate 6.09 mM (0.5 g/L). Total sugars 62.29 mM (10 g/L). Kinetic and stoichiometric parameters shown in supplementary tables were calculated at 36 h. [file 12934_2014_185_MOESM2_ESM.pdf]

Table S1. Kinetic parameters of growth strains in minimal medium with glucose 55.5 mM (10 g/L).

Strain	μ (h^{-1})	q_{Glc} (mmolC/ g_{DCW} h)	pHCA (μM)	$Y_{pHCA/Glc}$ (μmol_{pHCA}/ mmolC)	q_{pHCA} (μmol_{pHCA}/ g_{DCW} h)	CA (μM)	$Y_{CA/Glc}$ (μmol_{CA}/ mmolC)	q_{CA} (μmol_{CA}/g $_{DCW}$ h)	Acetic Acid (mM)
**WPJRg**	0.39 ± 0.05	62.04 ± 27.46	5.2 ± 0.65	0.05 ± 0.02	2.48 ± 0.88	13.2 ± 3.39	0.07 ± 0.03	4.40 ± 1.13	35.9 ± 11
**WPJAt**	0.28 ± 0.01	35.14 ± 2.89	---	---	---	527.68 ± 24.63	4.75 ± 0.17	197.09 ± 11.74	25.77 ± 12.42
**VPJRg**	0.15 ± 0.03	15.29 ± 4.44	107.21 ± 14.43	1.03 ± 0.56	27.62 ± 7.23	248.34 ± 56.25	1.08 ± 0.15	47.5 ± 2.6	11.9 ± 2.70
**VPJAt**	0.05 ± 0.01	11.10 ± 1.94	---	---	---	529.94 ± 33.25	17.01 ± 2.70	288.3 ± 47.9	---

Table S2. Kinetic parameters of growth strains in minimal medium with xylose 66.6 mM (10 g/L).

Strain	μ (h^{-1})	q_{Xyl} (mmolC/ g_{DCW} h)	pHCA (μM)	$Y_{pHCA/Xyl}$ ($μmol_{pHCA}$/ mmolC)	q_{pHCA} ($μmol_{pHCA}$/ g_{DCW} h)	CA (μM)	$Y_{CA/Xyl}$ ($μmol_{CA}$/ mmolC)	q_{CA} ($μmol_{CA}$/g $_{DCW}$ h)	Acetic Acid (mM))
**WPJRg**	0.41 ± 0.02	78.43 ± 14.38	25.4 ± 7.56	0.23 ± 0.12	16.02 ± 7.48	48.81 ± 5.53	0.42 ± 0.03	24.50 ± 2.27	21.87 ± 5.37
**WPJAt**	0.28 ± 0.02	28.10 ± 2.33	---	---	---	736.85 ± 52.11	6.03 ± 1.26	165.61 ± 3.86	11.01 ± 3.96
**VPJRg**	0.40 ± 0.02	59.13 ± 7.67	15.5 ± 12.24	0.14 ± 0.04	8.37 ± 3.50	24.35 ± 4.01	0.21 ± 0.05	12.18 ± 1.71	28.29± 7.83
**VPJAt**	0.24 ± 0.01	25.53 ± 3.13	---	---	---	315.98 ± 62.97	3.57 ± 0.61	139.0 ± 17.4	10.81 ± 5.73

Table S3. Kinetic parameters of growth strains in minimal medium with arabinose 66.6 mM (10 g/L).

Strain	μ (h^{-1})	q_{Ara} (mmolC/ g_{DCW} h)	pHCA (μM)	$Y_{pHCA/Ara}$ (μmol$_{pHCA}$/ mmolC)	q_{pHCA} (μmol$_{pHCA}$/ g_{DCW} h)	CA (μM)	$Y_{CA/Ara}$ (μmol$_{CA}$/ mmolC)	q_{CA} (μmol$_{CA}$/g $_{DCW}$ h)	Acetic Acid (mM)
**WPJRg**	0.42 ± 0.02	65.85 ± 9.48	76.75 ± 20.16	0.437 ± 0.11	14.39 ± 2.84	100.21 ± 15.72	0.5 ± 0.08	29.08 ± 3.82	3.55 ± 0
**WPJAt**	0.23 ± 0.003	38.33 ± 1.58	---	---	---	1022.36 ± 56.8	7.91 ± 1.62	368.91 ± 45.74	5.48 ± 1.4
**VPJRg**	0.39 ± 0.06	65.33 ± 23.59	27.01 ± 5.2	0.20 ± 0.06	7.18 ± 3.45	40.08 ± 5.89	0.29 ± 0.06	18.08 ± 4.40	19.75 ± 5.01
**VPJAt**	0.08 ± 0.01	26.95 ± 5.0	---	---	---	329.31 ± 23.28	15.67 ± 8.06	282.58± 82.53	20.38 ± 3.02

Table S4. Kinetic parameters of cultures with engineered strains grown in a minimal medium with simulated hydrolysate (SH). Glucose 6.66 mM (1.2 g/L), xylose 53.3 mM (8 g/L), arabinose 5.33 mM (0.8 g/L) and acetate 6.09 mM (0.5 g/L). Total sugars 62.29 mM (10 g/L)

Strain	μ (h^{-1})	q_{SH} (mmolC/ g_{DCW} h)	pHCA (μM)	$Y_{pHCA/SH}$ (μmol_{pHCA}/ mmolC)	q_{pHCA} (μmol_{pHCA}/ g_{DCW} h)	CA (μM)	$Y_{CA/SH}$ (μmol_{CA}/ mmolC)	q_{CA} (μmol_{CA}/g_{DCW} h)	Acetic Acid (mM)
**WPJRg**	0.41 ± 0.05	64.46 ± 16.54	39.16 ± 1.65	0.29 ± 0.06	18.17 ± 2.53	43.06 ± 7.13	0.31 ± 0.08	19.81 ± 5.20	38.48 ± 7.7
**WPJAt**	0.18 ± 0.06	30.50 ± 15.80	---	---	---	398.36 ± 74.16	4.73 ± 1.54	137.32 ± 8.2	21.51 ± 4.67
**VPJRg**	0.40 ± 0.03	59.79 ± 10.84	26.44 ± 9.4	0.20 ± 0.06	12.10 ± 4.20	44.27 ± 6.9	0.41 ± 0.09	24.03 ± 0.86	45.07 ± 9.95
**VPJAt**	0.17 ± 0.03	29.63 ± 6.59	---	---	---	260.49 ± 8.11	4.39 ± 2.00	180.52 ± 37.92	21.79 ± 4.7
**W(*pheA*$^-$)Rg**	0.14 ± 0.01	22.18 ± 3.51	322.9 ± 18.32	2.37 ± 0.13	52.57 ± 6.84	111.52 ± 7.9	0.460 ± 0.02	22.87 ± 1.61	25.18 ± 3.98
**W(*pheA*$^-$)At**	0.024± 0.003	14.17 ± 1.70	---	---	---	338.65 ± 33.08	3.65 ± 0.16	69.62 ± 7.43	13.99 ± 0.51
